# Supplementary material for: The genomic basis of host and vector specificity in non-pathogenic trypanosomatids
Source: Biol Open. 2022 May 3;11(4):bio059237. doi: 10.1242/bio.059237 (PMC9099014; doi:10.1242/bio.059237)
Supplement: Supplementary information [file biolopen-11-059237-s1.pdf]

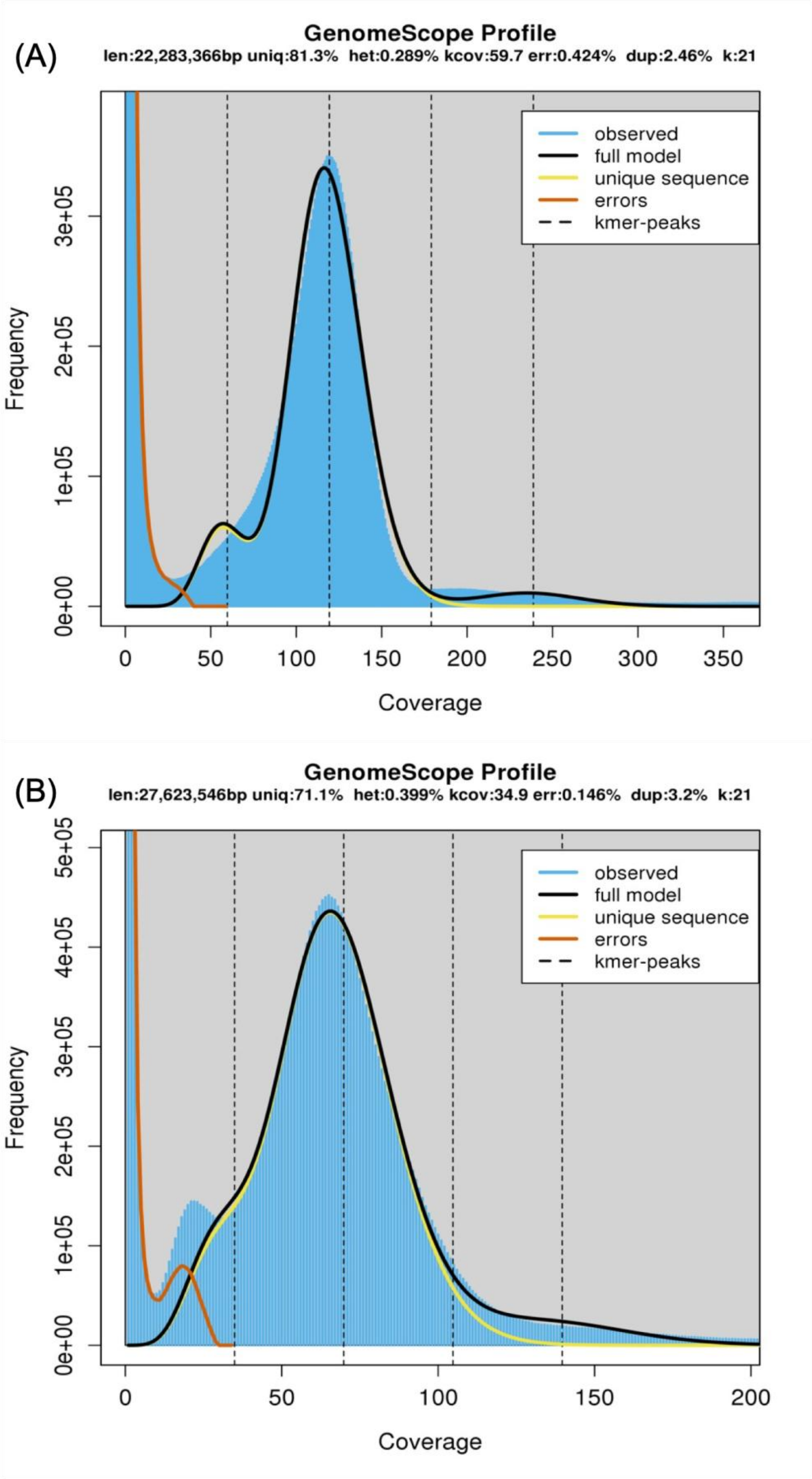

Fig. S1. k-mer spectra of (A) *T. melophagium* and (B) *T. theileri* using a k-mer size of 21. The statistics presented in these images represent the lowest estimate size, the highest estimate was used in Table 1 as these had a greater model fit.

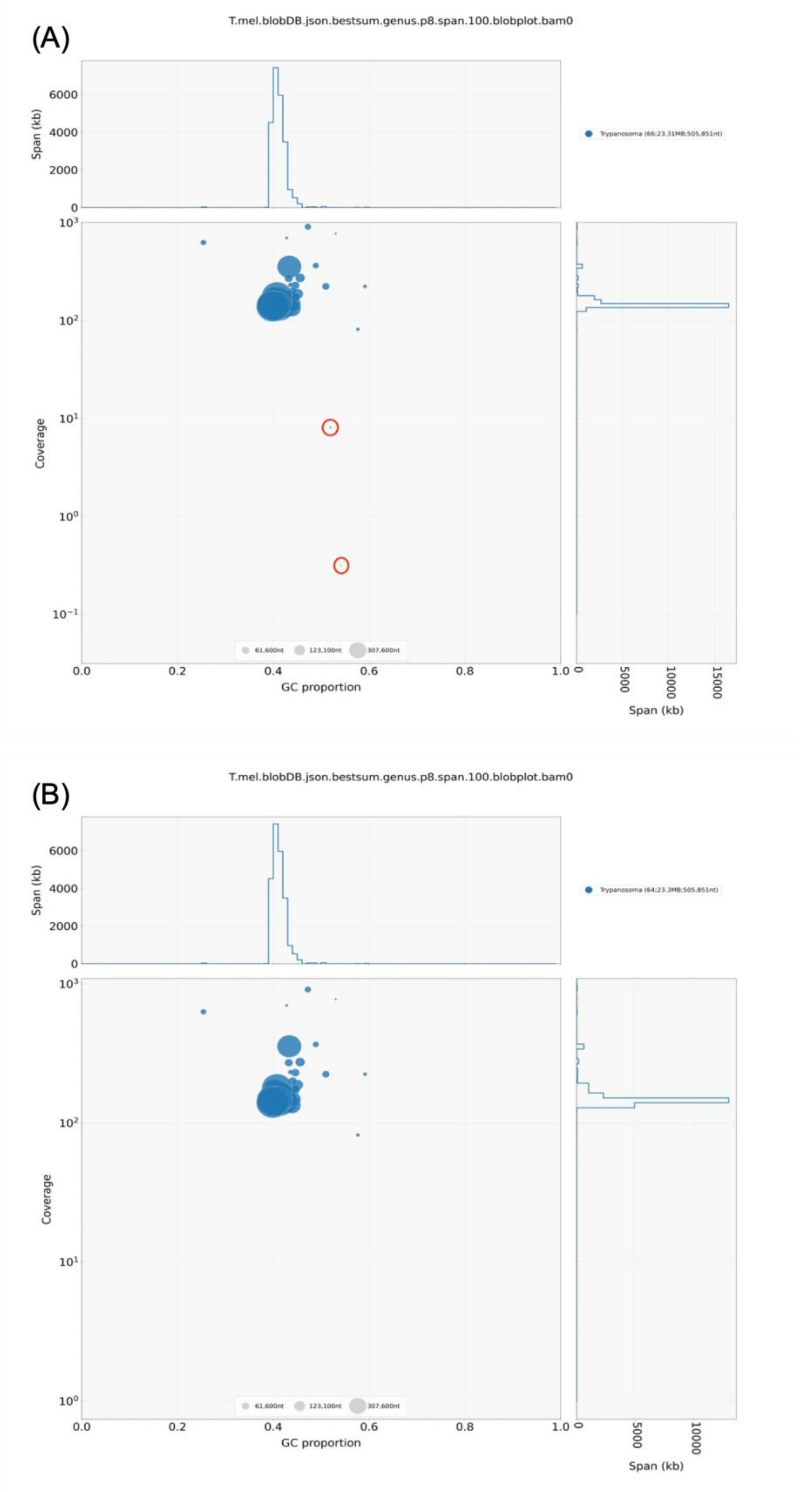

Fig. S2. Coverage and blast annotation for (A) the polished assembly and (B) the manually trimmed assembly.

Biology Open • Supplementary information

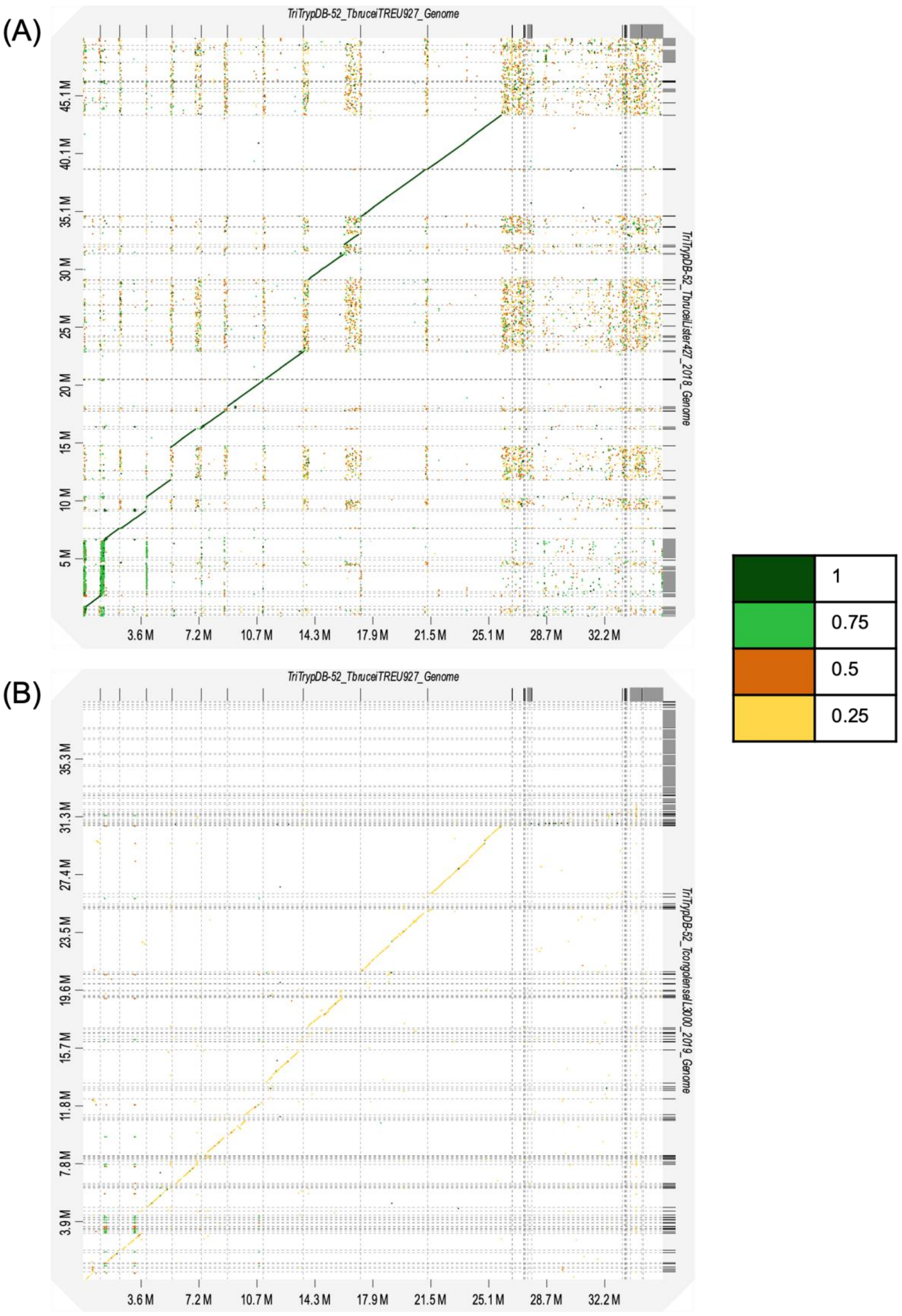

Fig. S3. Synteny plot of (A) *T. brucei* TREU927/4 and *T. brucei* Lister 427 (2018) and (B) *T. brucei* TREU927/4 *and T. congolense* IL3000 2019 genome sequences. The legend refers to the identity between the sequences.

Biology Open • Supplementary information

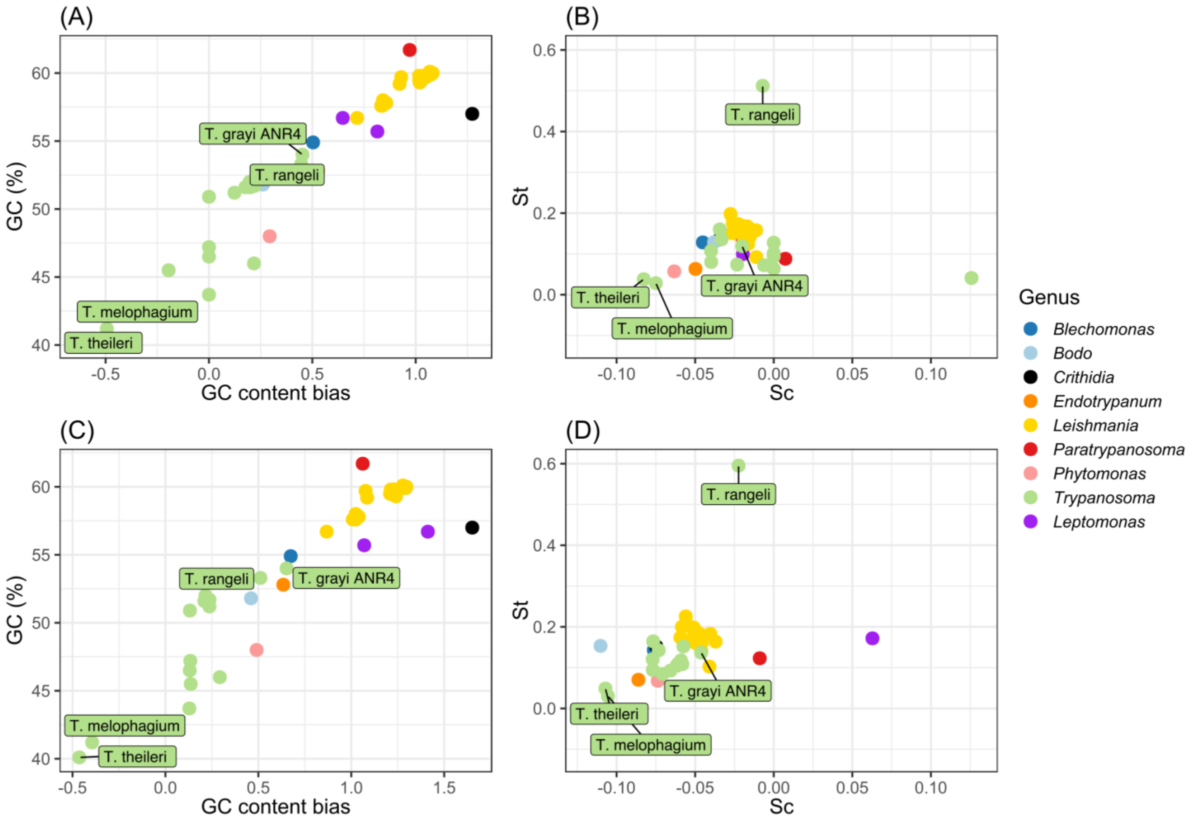

Fig. S4. (A) GC content across the whole genome and GC content bias in a every CDS. (B) Selection acting on translational efficiency (St) and selection acting on nucleotide cost (Sc). (C) GC content across the whole genome and GC content bias in CDS of a trypanosomatid universal single copy orthologue which is essential in every life cycle stage in *T. brucei* (n=158). (D) Selection acting on translational efficiency (St) and selection acting on nucleotide cost (Sc) in trypanosomatid universal single copy orthologues which is essential in every life cycle stage in *T. brucei* (n=158).

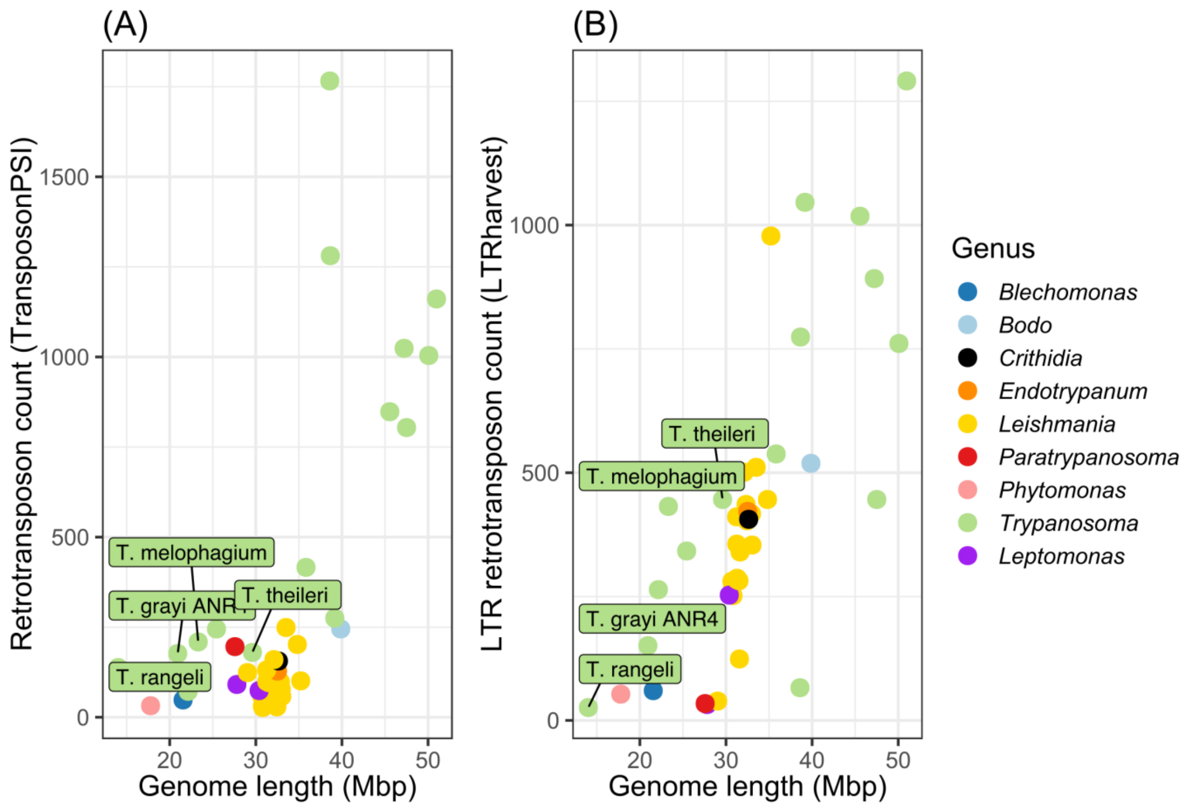

Fig. S5. (A) Retrotransposon (B) long terminal repeat (LTR) retrotransposon counts compared to genome length.

Biology Open • Supplementary information

Table S1. Software and any options used.

Tool	Version	Flag	Available at
**Guppy**	4.0.15	--flowcell FLO-MIN106<br>--kit SQK-RAD004	https://community.nanoporetech.com/
**PycoQC**	2.5.0.3		https://github.com/a-slide/pycoQC
**Trimmomatic**	0.39	-SLIDINGWINDOW 4:20<br>-MINLEN 50	https://github.com/timflutre/trimmomatic
**GenomeScope**	1	-Kmer 21<br>-Max kmer cov. 1000	http://qb.cshl.edu/genomescope/
**Jellyfish**	2.2.10	-C -m 21 -s 1000000000	https://github.com/gmarcais/Jellyfish
**Wtdbg2**	3	-x ont -g 30m -A -t 10	https://github.com/ruanjue/wtdbg2
**Minimap2**	2.17-r941	-ax map-ont	https://github.com/lh3/minimap2
**Racon**	v1.4.13	-m 8 -x -6 -g -8 -w 500	https://github.com/isovic/racon
**Medaka**	V5	-m r941_min_high_g360	https://github.com/nanoporetech/medaka
**BWA-MEM**	0.7.17-r1188		https://github.com/lh3/bwa
**Pilon**	1.24	--genome<br>--diploid –bam<br>--bam	https://github.com/broadinstitute/pilon
**Blast**	2.6.0+	blastn<br>-task megablast<br>-outfmt '6 qseqid	ftp://ftp.ncbi.nlm.nih.gov/blast/executables/blast+/LATEST/

Biology Open • Supplementary information

		staxids bitscore std'<br>-max_target_seqs 1<br>-max_hsps 1<br>-evalue 1e-25 or 1e-5	
**DIAMOND**	2.0.5.143	Blastx<br>--sensitive<br>--max-target-seqs 1<br>--evalue 1e-25	http://www.diamondsearch.org
**BlobTools**	1.1.1	-r genus	https://github.com/DRL/blobtools
**Scaffold_stats.pl**	N/A		https://github.com/sujaikumar/assemblage
**BUSCO**	V5	-l euglenozoa_odb10	https://busco.ezlab.org/
**RepeatModeler**	N/A	-database -LTRStruct -pa 8	http://www.repeatmasker.org/RepeatModeler/
**RepeatMasker**	N/A	-lib consensi.fa.classified -pa 8 -xsmall -nolow –gff	http://www.repeatmasker.org/RMDownload.html
**BRAKER2**	2.1.6	--prot_seq<br>--bam<br>--softmasking<br>--etpmode<br>--gff3	https://github.com/Gaius-Augustus/BRAKER
**D-Genies**	1.2.0	-Minimap2	http://dgenies.toulouse.inra.fr/run
**tRNAscan-SE**	2.0.9	-E	http://lowelab.ucsc.edu/tRNAscan-SE/
**CodonMuse**	0.1.0	-f<br>-tscan	https://github.com/easeward/CodonMuSe

Biology Open • Supplementary information

**TransposonPSI**	1.0.0	-nuc	http://transposonpsi.sourceforge.net
**gt suffixerator**	1.6.2	-tis -suf -lcp -des -ssp -sds -dna	http://genometools.org/tools/gt_ltrharvest.html
**gt LTRharvest**	1.6.2		http://genometools.org/tools/gt_ltrharvest.html
**OrthoFinder**	2.5.2	-S diamond_ultra_sens	https://github.com/davidemms/OrthoFinder
**InterProScan**	5.52-86.0	--dp<br>--goterms<br>-appl SignalP-EUK-4.1, Pfam<br>-f TSV	https://www.ebi.ac.uk/interpro/
**KinFin**	1.0.3		https://github.com/DRL/kinfin
**iTOL**	6		https://itol.embl.de
**R**	3.6.1		https://www.r-project.org/
**ggplot2**	3.3.3		https://cran.r-project.org/web/packages/ggplot2/index.html
**ggrepel**	0.9.1		https://cran.r-project.org/web/packages/ggrepel/index.html

Biology Open • Supplementary information

Table S2.

Click here to download Table S2

Table S3.

Click here to download Table S3

Table S4.

Click here to download Table S4

Table S5.

Click here to download Table S5
